# Colorectal cancer treatment using bacteria: focus on molecular mechanisms

**DOI:** 10.1186/s12866-021-02274-3

**Published:** 2021-07-19

**Authors:** Sara Ebrahimzadeh, Hossein Ahangari, Alireza Soleimanian, Kamran Hosseini, Vida Ebrahimi, Tohid Ghasemnejad, Saiedeh Razi Soofiyani, Vahideh Tarhriz, Shirin Eyvazi

**Affiliations:** 1 Department of Pharmaceutical Biotechnology, Faculty of Pharmacy, Tabriz University of Medical Sciences, Tabriz, Iran; 2 Department of Food Science and Technology, Faculty of Nutrition and Food Science, Tabriz University of Medical Sciences, Tabriz, Iran; 3 Department of Biology, Faculty of Natural Sciences, University of Tabriz, Tabriz, Iran; 4 Molecular Medicine Research Center, Biomedicine Institute, Tabriz University of Medical Sciences, Tabriz, Iran; 5 Department of Pharmaceutical Biotechnology, School of Pharmacy, Shahid Beheshti University of Medical Sciences, Tehran, Iran; 6 Clinical Research Development Unit of Sina Educational, Research and Treatment Center, Tabriz University of Medical Sciences, Tabriz, Iran; 7 Department of Biology, Tabriz Branch, Islamic Azad University, Tabriz, Iran; 8 Biotechnology Research Center, Tabriz Branch, Islamic Azad University, Tabriz, Iran

**Keywords:** Colorectal cancer, Biotherapeutical toxins, Bacteriocins, Bacterial peptides

## Abstract

**Background:**

Colorectal cancer which is related to genetic and environmental risk factors, is among the most prevalent life-threatening cancers. Although several pathogenic bacteria are associated with colorectal cancer etiology, some others are considered as highly selective therapeutic agents in colorectal cancer. Nowadays, researchers are concentrating on bacteriotherapy as a novel effective therapeutic method with fewer or no side effects to pay the way of cancer therapy. The introduction of advanced and successful strategies in bacterial colorectal cancer therapy could be useful to identify new promising treatment strategies for colorectal cancer patients.

**Main text:**

In this article, we scrutinized the beneficial effects of bacterial therapy in colorectal cancer amelioration focusing on different strategies to use a complete bacterial cell or bacterial-related biotherapeutics including toxins, bacteriocins, and other bacterial peptides and proteins. In addition, the utilization of bacteria as carriers for gene delivery or other known active ingredients in colorectal cancer therapy are reviewed and ultimately, the molecular mechanisms targeted by the bacterial treatment in the colorectal cancer tumors are detailed.

**Conclusions:**

Application of the bacterial instrument in cancer treatment is on its way through becoming a promising method of colorectal cancer targeted therapy with numerous successful studies and may someday be a practical strategy for cancer treatment, particularly colorectal cancer.

## Introduction

Colorectal cancer (CRC) is a serious disease characterized as uncontrolled division or abnormal growth of colon or rectum cells. CRC is the second major cause of cancer-related global mortality [1]. In the United States, almost close to 150,000 new cases of CRC are identified per annum. However, the greatest prevalence has been reported from Australia, New Zealand, Europe, and North America, while the lowest rates have been reported from Africa and South-Central Asia [2]. The risk of developing CRC is affected by genetic, epigenetic and environmental factors [3]. Genetic risk factors such as Type2 diabetes, family history of CRC, and history of inflammatory bowel disease (IBD) can increase the risk of CRC in a long period. The utilization of anti-inflammatory medications for the treatment of IBD considerably reduces the risk of CRC incidence [4, 5]. Nevertheless, the great portion of CRC cases has been associated with environmental factor such as a sedentary lifestyle, unhealthy diet, smoking, and obesity. Besides the aforementioned risk factors, the pathogenic microorganisms can also play a critical role to develop CRC [6]. The large intestine is an organ continuously exposed to bacteria like *Fusobacterium* sp.*, Porphyromonas*, *Escherichia coli, Helicobacter pylori*, *Citrobacter rodentium, Bacteroidetes,* and *Prevotella*, which are all reported prevalently in the biopsies of patients with CRC [7–10].

There are several conventional CRC treatment strategies starting from simple endoscopic polypectomy to wider surgical, to radio-chemotherapy combinations and end in complicated chemotherapeutical regimens mixed with drugs. However, these treatment strategies all have their own drawbacks and side effects [11]. Studies for finding highly selective treatments have indicated that although bacteria account for a large number of cancers, most of them have antimicrobial and antifungal activities, which can be used in cancer therapy [12–14]. William B. Coley who used combination of *Serratia marcescens* initially presented cancer therapy using bacteria in the late nineteenth century and *Streptococcus pyogenes* to treat sarcomas, witnessed tumor abatement, and increased patient survival. After his novel discovery, numerous studies have shown phenomenal results in using various bacteria in the eradication of different tumors. In the current study, the recent advances in CRC therapy utilizing bacteria are presented and focused on the molecular mechanisms that are targeted by the bacterial treatment in the CRC tumors. Introduction of advanced and successful strategies in bacterial CRC therapy could be useful to identify novel promising treatment strategies for CRC patients.

### Bacterial cancer therapy

Cancer is a serious debilitating cause of death in recent decades that has enforced scientists to discover new prevention and treatment methods. Among the methods, bacterial therapy is one of the attractive strategies. Although good results were obtained from these treatments, conversely there were qualms due to the bacterial infections [15]. A safe bacterium-mediated cancer therapy should have features such as cancer cytotoxicity or immunogenicity, reducing toxicity to intact cells, cancer selectivity as well as stability in the human body conditions. The secretion of cancer cytotoxic substances by the different bacterial strains has been widely reported, whereas so far, the knowledge about responsible genes of these secreting substances is challenging [10]. According to the confirmation of the safety of bacterial cancer therapy, researchers have used *Clostridia* and *Streptococci* species for this purpose in the previous research, but nowadays, the main focus is on the genetically modified species owing to their greater capability of binding to cancer cells [16, 17]. *Bifidobacteria* sp., *Salmonellae* sp. and *Clostridia* sp. are the common species that have been tested in animal models bearing various tumors. The bacteria act as a vector for the delivery a wide range of genes such as anti-angiogenic genes, apoptosis genes, tumor suppressor genes, and tumor-linked antigens [18, 19]. Genetically modified bacteria can be expended in the acceleration of cancer detection as dual diagnostic and medicinal instruments. In this regard, a large number of studies have discovered that genetically modified bacteria can have a more significant multiplication in tumors than in normal tissues [20]. Cytotoxicity of bacteria in various tumor cells and their secreted substances are listed in Table 1.

**Table 1:** Bacterial strains with cancer cytotoxic traits

Bacteria	Substance	Cancer type	Reference
*Streptomyces fradiae*	L-asparaginases	Colorectal Adenocarcinoma	[21]
*Brevibacillus* spp.	Laterosporulin 10	Breast cancer, embryonic kidney cancer	[22]
*Streptomyces albulus*	L-lysin (ε-PL)	Hepatocellular carcinoma	[23]
*Enterobacter cloacae*	L-asparaginases	Leukemia	[24]
*Corynebacterium Diphtheriae*	Diphtheria toxin	Adrenocortical carcinoma, cutaneous T cell lymphomas	[25]
*Pseudomonas aeruginosa, Sphingobacterium* spp.	Arginine deiminase	Prostate carcinoma, colon adenocarcinoma	[26]
*Serratia surfactantfaciens*	Serrawettin W2	Cervical carcinoma	[27]
*Clostridium novyi*	Phospholipases	Colon carcinomas, sarcoma	[28]

### Colorectal cancer therapy using bacteria

**Fig. 1:**
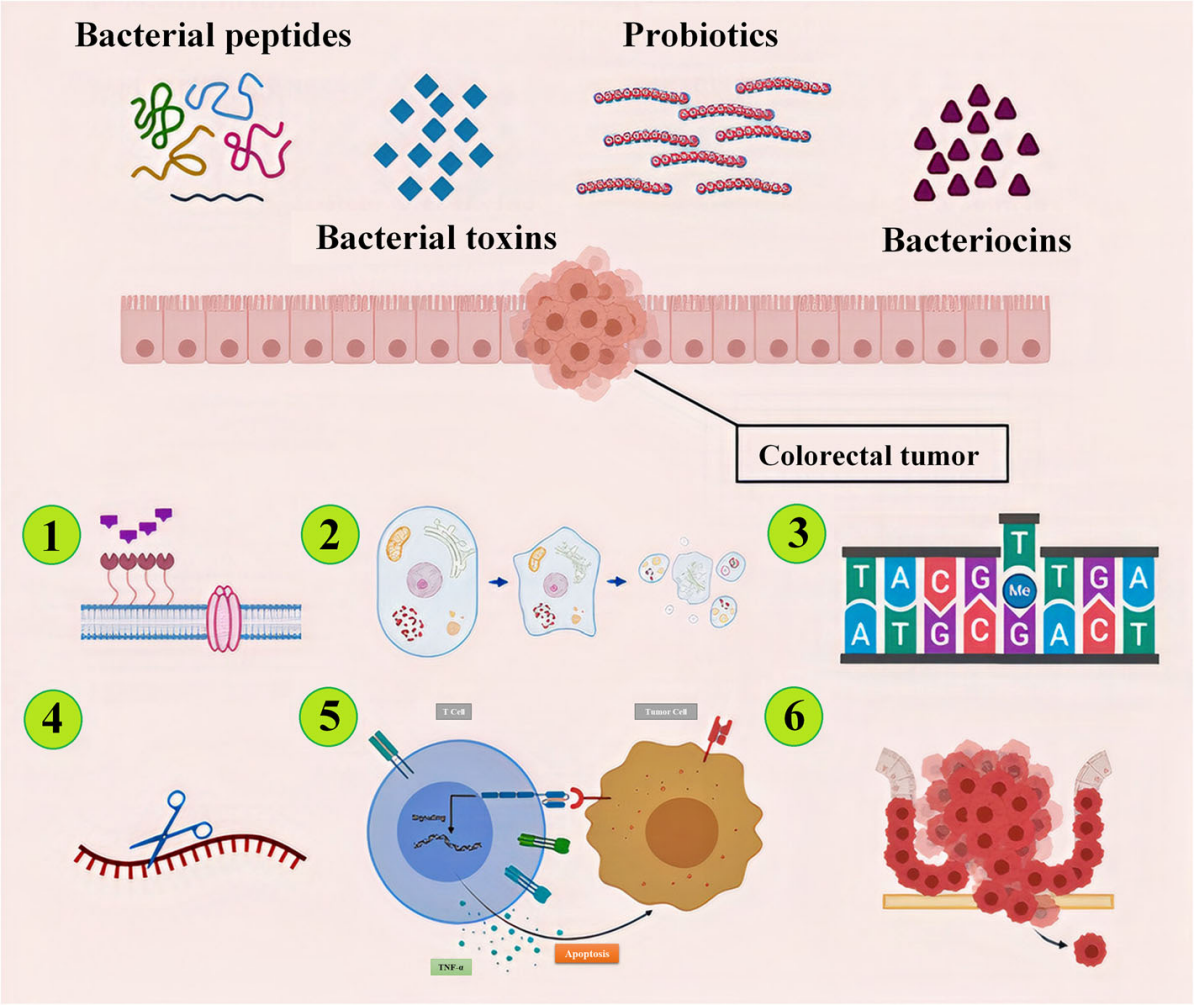
The colorectal cancer bacterial therapy can be performed by means of bacterial whole cell such as probiotics or bacterial-associated peptides like bacteriocins or bacterial toxins. The anticancer effect of this treatment can be achieved by different mechanisms: 1) Pore forming in the cell membrane 2) Induction of apoptosis 3) DNA alkylation 4) RNase activity 5) TNF-α production 6) Inhibition of metastasis [10, 29]

Using bacteria for the treatment of CRC has become an important issue for many researchers and various mechanisms have been recognized for bacteria’s role in CRC treatment (Fig. 1). Toxins secreted from bacterial are the other effective therapeutic agents that have been utilized for CRC therapy. It has been shown that bacterial peptides are the potent agents in CRC therapy. In addition, secreted substances from bacteria which are used as a carrier and combining them with medications to targeted delivery of some anticancer medications to cancerous cells is another method using bacteria in CRC treatment [10]. For example, in recent published research, 5-Fluorouracil (5-FU) resistant CRC cells have shown that they are responsive to the combination of 5-FU and *Lactobacillus plantarum;* consequently, the combination resulted in further anti-cancer activity for cells. However, the role of bacteria in the administration of probiotics as a complementary treatment to combat CRC should not be overlooked [29].

### Bacteriocins for CRC therapy

Bacteriocins are antibacterial peptides synthesized by many bacteria’s ribosomes to inhibit the growth of other bacterial strains. Some of these bacteriocins are reported with anti-cancer features [30]. Several bacteriocins that have reported efficacious tumoricidal activity on CRC are nisin, colicin, microcin and pediocin. Nisin is a positive charged molecule that causes pore development in the cell membrane of the target organism and thereby results in cytoplasmic membrane depolarization [31]. Nisin is synthesized and secreted by *Lactococcus lactis* appeared to have a considerable cytotoxic effect against CRC cell lines such as Caco-2 and HT-29 colorectal cancer cell lines [32]. In a study that the apoptotic effect of nisin on the SW480 cells was investigated, cell viability and Bax/Bcl-2 ratio at both mRNA and protein levels were considered. It has been shown that the doses of 2000–4000 μg/ml of nisin had a significant anti-proliferative impact on SW480 cell line and also could increase the apoptotic index (*p* < 0.05) [33]. Apart from anti-proliferative feature of nisin, it is known to have a potential preventing effect against the expression of metastatic genes such as MMP2, MMP9, cytolethal distending toxins (CDTs) and the cycle inhibiting factor (Cif), and up-regulates the expression of the genes inhibitors in CRC cell lines such as LS-180, HT-29, SW480, and Caco-2 [34].

Data showed that the expression level of carcinoembryonic antigen (CEA) is associated with metastasis of CRC cells. Norouzi et al., (2018) showed that nisin decreased the CEA level in condition media of colorectal cancer cell lines [34]. One of the mentioned roles of nisin is the enhancing the cytotoxicity of some chemotherapeutics when used as a combination. Furthermore, nisin was shown to be a cytotoxic agent that acts as a cell membrane disrupter and an apoptotic pathway activator. This function can be represented alone or in combination with chemotherapeutic agents such as Etoposide, 5-FU, and Hydroxyurea. It has been reported that the combination of nisin with conventional therapies lowered the therapeutic doses of these anti-cancer drugs [35]. Colicin is another bacteriocin released from *Enterobacteriaceae* (e.g., *E. coli*) that inhibits the growth of other bacteria and lessens the competition. Available evidence shows that the cytotoxic effect of different colicins on various cancerous cells are likely through pore-forming, a non-specified DNase activity, RNase activity, and inhibitory effect on murein biosynthesis [36]. In this way, the cytotoxic effect of different colicins has been studied in vitro on HT-29 cell lines (a type of human colon cancer cell line) [37]. Going into depth, in this study, the growth inhibitory effect of colicins E1, E3, A, and U on 11 human cancer cell lines, in addition to HT-29, was explored. Results revealed that HT-29 colon carcinoma cells are insensitive in response to colicin E1 and the highest cytotoxicity against HT-29 was achieved by colicin A treatment [37]. However, it was not mentioned that Colicin has more toxicity to tumor cells in comparison to the normal cells [38]. For instance, microcin E492 is a part of *Klebsiella Pneumoniae. coli*cins have a cytotoxic effect against colorectal carcinoma cells while there is not any reaction against the normal cells [10]. Besides, comparing the anti-tumorigenic effect of microcin E492 on two different cell lines (HT-29 and SW620) in both in vitro and in vivo showed that microcin E492 has more cytotoxicity effect on the HT-29 cell line compared with SW620. The impact is believed to happen by the mechanism of apoptosis. For in vivo investigation, SW620 zebrafish larvae xenografts were developed. The intratumoral injection of microcin E492 reduced tumor growth significantly [32]. Studies indicated microcin depolarizes cell membrane potential by pore-forming ability, and also DNA fragmentation, phosphatidylserine release, caspase activity, and releasing of intracellular calcium ions are the principle mechanisms of apoptotic cell death by inducing microcin [39]. Pediocin is another bacteriocin that has cytotoxic effects on CRC cells such as HT-29 and DLD-1. The toxin is secreted by *Pediococcus acidilactici* (K2a2–3) which is isolated from the intestine of Philippine water buffalo [40]. The different important bacteriocins, which can be used in CRC therapy, are summarized in Table 2.

**Table 2:** Bacteriocins in colorectal cancer therapy

Bacteriocin	Producer microorganism	Molecular weight (kda)	Affected Cell lines	Reference
Nicin	*Lactococcus lactis*	3.5	Caco-2, HT-29, LS-180, SW48	[41]
Colicin	*Enterobacteriaceae* (e.g. *E. coli*)	4.1	HT-29	[36]
Microcin E492	*Klebsiella pneumoniae*	7.8	CRC cells, HT-29, SW620	[32]
Pediocin	*Pediococcus acidilactici* K2a2–3	6	HT-29, DLD-1	[40]

### Bacterial toxins in CRC therapy

Toxins and other bacterial components have been studied since they have a crucial role as anti-cancer substances. Numerous investigated evidence show that bacterial toxins act as a valuable inhibitor of cancerous cell growth. Bacterial toxins have a twofold manner on cancer cells based on the concentration. In other words, they can cause cell death or amplify the cell’s proliferation. To shed more light on this issue, in high levels, they can lyse these cells whereas in low concentrations they can modify the cellular procedures which are responsible for proliferation and apoptosis regulation [42]. Two different anti-cancer toxins have been recognized in bacteria which include toxins with the ability to conjugate on surface antigens of cancerous cells and toxins which can conjugate the ligands of cancer cells. Cyclomodulins refer to bacterial toxins that stimulate or block the eukaryotic cell cycle. In addition, cytotoxic necrotizing factor (CNF) is another toxin released by certain bacterial strains like *E. coli* K-12. CNF motivates the transition of G1-S cycle as well as DNA duplication, which causes the cells to become multinucleated without any changes in the total number of cells. This is probably due to the toxin capability preventing cell differentiation and activating cell apoptosis [43].

Generally, CRC cells have numerous tumor-specific antigens on their surface and most of them act as receptors. Several toxins like diphtheria toxin (DT) become activated after binding to these receptors [44]. The toxins can act as a ligand for binding to selective receptors on the target cell [45]. We briefly described some bacterial toxins that have been used in CRC therapy studies. One of the successful toxins in CRC therapy is *enterotoxin* (CPE) produced by *C. perfringens*. It has been shown that CPE exactly attaches to claudin-3 and claudin-4 which abundantly exist on the cancerous cell surface. This complex is known as a “multi-protein membrane pore complex” and causes the lysis of cancerous cells through losing cellular osmotic equilibrium. The anti-cancer feature of CEP for CRC cells was confirmed in in vivo and in vitro studies [46, 47]. For instance, Pahle et al.*,* (2017) verified an optimized CPE expressing vector as a target for claudin-3 and/or claudin-4 expressing in colon cancer cells including SW-480, HCT-116, SW620, Caco-2, HT-29 and PDX (patient-derived colon carcinoma xenografts). The results showed that the CPE is a gene transfer system and can be considered as an appealing therapeutic agent in colon carcinomas through the targeting of claudin-3 and/or claudin-4 which lead to rapid and impressive tumor cell killing in both in vitro and in vivo conditions [46]. On the molecular scale, the transfection of optCPE directs rapid cytotoxic effects including necrosis in claudin expressing cells due to the membrane breakdown. On the other hand, massive necrosis and decrease in tumor cell growth in colon carcinoma PDX bearing mice were detected because of the intratumoral optCPE expression. Therefore, it was revealed that optCPE gene transfer causes lysis in claudin-positive tumor cells, while claudin-negative cells stayed unchanged. Szeponik et al., demonstrated that DT effect on regulatory T cells (T_reg_) depletion and its effect on the density as well as of on and effector action of various TCR^αβ+^ and TCR^γδ+^ T cell populations in intestinal tumors. In this study, the APC^Min/+^\DEREG mouse model has used which ports a DT receptor under the control of the FOXP3 promoter to deplete T_reg_ in mice suffering CRC. They have indicated that the density of conventional TCR^αβ+^ CD8^αβ+^ T cells was meaningfully increased in T_reg_ depleted tumors and T cells showed improved activation and proliferation in addition to increased IFN-γ and Granzyme B production. Investigating the molecular mechanisms via immunohistochemistry staining and flow cytometry demonstrated a noteworthy proliferation of CD8αβ T cells in the T_reg_-depleted tumors [48]. Maslowski et al.*,* (2019) depleted a *Salmonella enterica* serovar Typhimurium (STm) in mice CRC cells for evaluation of the mechanisms and efficiency of medication they employed an Aromatase A-deficient STm (STm^ΔaroA^). STm^ΔaroA^ which was delivered by oral gavage showed that it has the ability to meaningfully decrease the tumor load in intestinal cancer models, Apc^min/+^ mice. The colonization of STm^ΔaroA^ in tumor cells causes the modification in the mRNA transcription which correlated with the epithelial-mesenchymal transition, cell cycle-related transcripts and metabolic. The analysis of metabolomics in tumor cells confirmed the changes in the metabolic condition of STm^ΔaroA^ treated tumors, revealing that STm^ΔaroA^ inflicts some metabolic antagonism on the tumor cells. In conclusion, the STm^ΔaroA^ in in vitro tumor organoid condition has direct effect on tumor epithelium and results to change in transcripts and metabolites which is similar to what happens in vivo-treated tumors [49].

### Bacterial peptides in CRC therapy

Using biomaterial is another effective agent in cancer therapy, which has a profound effect on tumor cells as an anti-proliferative agent. Because they can activate lymphocytes and macrophages as well as induce the production of tumor necrosis factor α (TNF-α) as a cytotoxic substance, are activated by microbial infections [50]. These types of peptides are characterized by hydrophobicity and low molecular weight (in the range of 3 kDa to 10 kDa) that are very vital for their penetration into the cancer cells to inhibit their growth. Several types of bacterial peptides were demonstrated that can arrest cell cycle progresses while some of them can induce apoptosis. Furthermore, some types of bacterial peptides are better than chemotherapy drugs due to the fact that using bacterial peptides reduce side effects or even don’t have any side effect. To name but few some of these kinds of peptides such as azurin, pediocin K2a2–3, mitomycin C, Enterococcal anti-proliferative peptide (Entap), and Nonribosomal Peptides (NRPs) can be used by clinicians in the cancer treatment process (Table 3).

**Table 3:** Some of the important bacterial peptides that have been used for CRC therapy

Peptide/Protein	Source	References
Azurin	*Pseudomonas aeruginosa*	[25, 51, 52]
Pediocin K2a2–3	*Pediococcus acidilactici* K2a2–3	[40]
Mitomycin C	*Streptomyces caespitosus*	[10]
Entap	*Enterococcus* sp.	[32]
Lucentamycin A and B	*Nocardiopsis lucentensis* CNR-712	[53]
Arenamide A and B	*Salinispora arenicola*	[54]
Ohmyungsamycins	*Streptomyces* sp.	[55]
Mixirins	*Bacillus* sp.	[56]
Urukthapelstatin A	*Mechercharimyces asporophorigenens* YM11–542	[57]

#### Azurin

Azurin is a kind of copper containing protein secreted by *P. aeruginosa*. The protein has anti-cancer activity when present in blood [58, 59]. The anti-cancer activity is related to several mechanisms such as forming complexes with p53 tumor suppressor, interferential activity on the receptor of tyrosine kinase EphB2-mediated signaling process, reducing of activity of VEGFR-2 tyrosine kinase, preventing of angiogenesis, and interferential activity on P-cadherin protein expression [25]. Azurin operates as an electron transfer shuttle in bacterial species. According to mentioned azurin anti-cancer mechanisms, complex forming between p53 and azurin can help p53 to be stable and improve its function by inhibiting COP1-mediated ubiquitination, and causes proteasome degradation, therefore p53 induces cell cycle arrest at G2/M and this inhibits the cancer development. Some kinds of biological assays such as apoptosis assay (by using flow cytometry) and cytotoxicity assay (like MTT assay) approve these functions [51]. Other cancer cell lines that azurin can exert its cytotoxic role on them are oral squamous carcinoma (YD-9), breast cancer (MCF-7, MDA-MB-157), and melanoma (UISO-Mel-2) [60].

#### Pediocin K2a2–3

Pediocin K2a2–3 was first isolated from *Pediococcus acidilactici* K2a2–3 and has shown an effective anti-cancer role [31, 61, 62]. Reference to previous research [40], pediocin K2a2–3 found to be cytotoxic against HT-29 colon adenocarcinoma cells because of hydrophobicity of this peptide based on MTT assay. The hydrophobic nature of this peptide may be the reason for its cytotoxicity. This high hydrophobicity pediocin was an advantage for the first step purification process of it from the supernatant of strain overnight culture. After partial purification, the pH-mediated method was utilized to obtain dialyzed and undialyzed extracts [40].

#### Mitomycin C

Mitomycin C is a kind of antibiotic that is used as anti-cancer material and was isolated from *Streptomyces caespitosus*. The molecular weight and molecular formula of mitomycin C are 334 Da and C_15_H_18_N_4_O_5_. This antibiotic is used in the treatment of some cancers such as colorectal, anal carcinomas, lungs, head, neck, and breast cancer. This kind of anti-tumor antibiotic binds to DNA on the alkylation and inhibits DNA synthesis [10]. An enzymatic bio-reduction requires for the reduction of mitomycin C and its activation. After reduction, a very reactive bis-electrophilic is formed that alkylates cellular nucleophiles which is the main mechanism of mitomycin C. Moreover, the cytotoxic effects of mitomycin C is related to the formation of DNA-DNA interactions [10]. Therefore, it has a very critical role in the treatment of cancers.

#### Enterococcal anti-proliferative peptide (Entap)

Another type of bacterial peptide that has anti-proliferative activity is Entap. This type of peptide produced by *Enterococcus* sp*.* and arrest cancer cells in G1 and stimulation of autophagic apoptosis. Cancer cell studies demonstrated numerous vacuoles and autolysosomes and autophagosomes structures in the cells. Entap has anti-proliferative activity against prostatic carcinoma (22Rv1), mammary gland adenocarcinoma (MDA-MB-231), human gastric adenocarcinoma (AGS), uterine cervix adenocarcinoma (HeLa), and colorectal adenocarcinoma (HT-29) cell lines [32].

#### Nonribosomal peptides (NRPs)

Bacteria, fungi, and cyanobacteria can synthesize Nonribosomal peptides (NRPs) and bioactive metabolites which have anti-cancer activity [63]. Some of NRPs have an anti-colorectal activity, which include lucentamycins, arenamides, ohmyungsamycins, mixirins, and urukthapelstatin A.

#### Lucentamycins

**Fig. 2:**
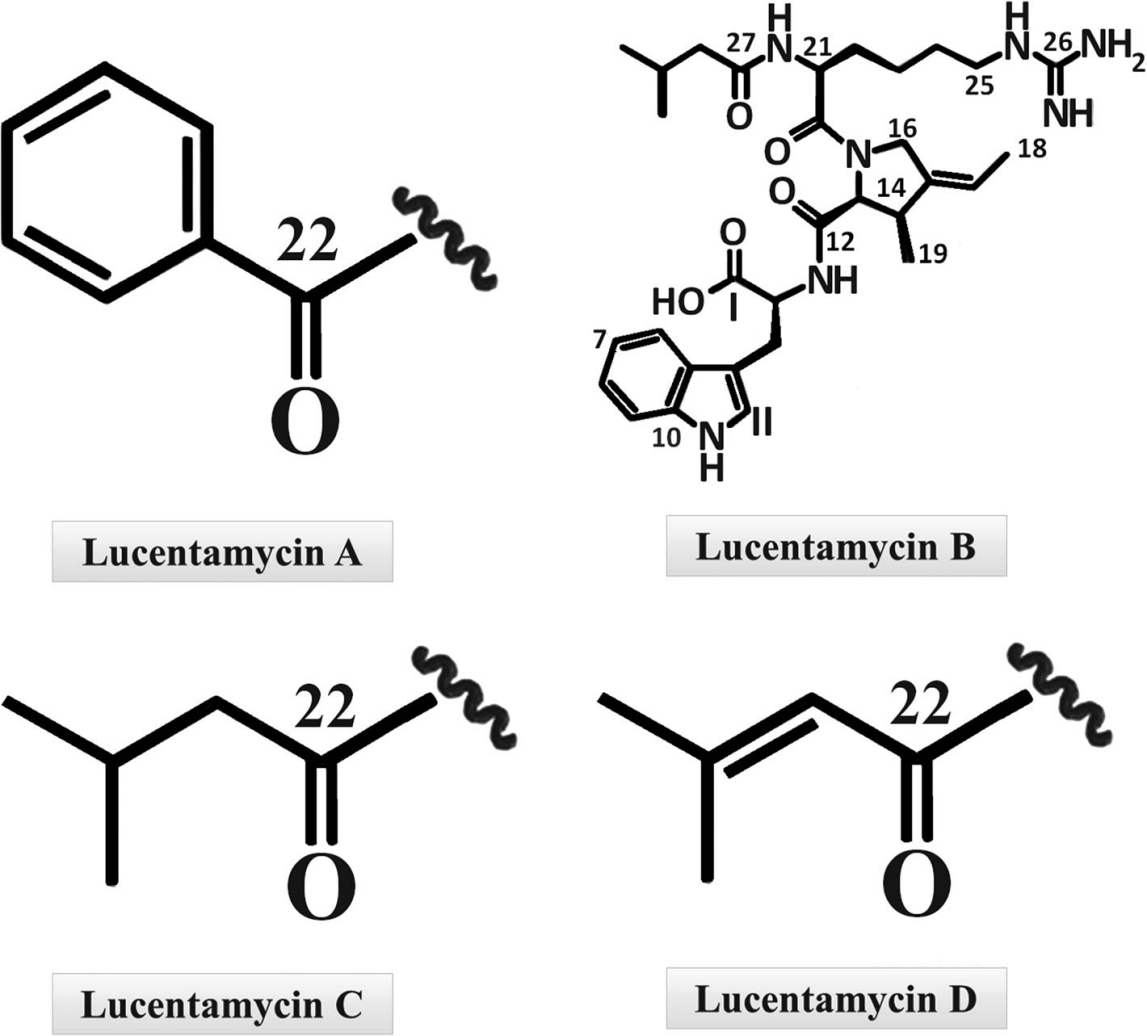
Four different structure of Lucentamycins including Lucentamycins A, B, C and D [53]

Lucentamycins (A-D), 3-methyl-4-ethylideneproline-containing peptides are type of cytotoxic peptides isolating from the crude extract of *Nocardiopsis lucentensis* CNR-712. Four structurally different lucentamycins (Fig. 2) were isolated by culture extract fractionation. After the structure determination of each compound by NMR technique, the bioassay test (3-(4,5-dimethylthiazol-2-yl)-5-(3-carboxymethoxyphenyl)-2-(4-sulfophenyl)-2H-tetrazolium (MTS) assay) was performed to measure cytotoxic activity against HCT-116 colon carcinoma cell line. The in vitro cytotoxicity of lucentamycins A and B was significant with the IC50 of 0.2 μM and 11 μM [53].

#### Arenamides

*Salinispora arenicola* secreted new types of cyclohexadepsipeptides (arenamides A to C) in sea sediment. Previous research have shown that TNF-induced activation is blocked by arenamides A and B. These kind of arenamides have important roles in inhibition of nitric oxide and prostaglandin E2 production and also moderate cytotoxic effect on human colon carcinoma (HCT-116) [54].

#### Ohmyungsamycins

Cyclic peptides ohmyungsamycin A and B were secreted by *Streptomyces* sp. which both of them are comprise of amino acid units, such as N,N-dimethylvaline, β-hydroxyphenylalanine, and N-methyl-4-methoxytrytophan. Growth inhibition and anti-proliferative activity against various cancerous cell lines were demonstrated by ohmyungsamycin A and B [55].

#### Mixirins

Three kinds of cyclic acylpeptides (mixirins A to C) are secreted by the marine bacterium *Bacillus* sp. Their formula are C_48_H_75_N_12_O_14_, C_45_H_69_N_12_O_14_ and C_47_H_73_N_12_O_14_, respectively, and the molecular weight of them are about 1 KDa. All of the mixirins (A, B, and C) can block the growth of HCT-116 (human colon tumor cell line) [56].

#### Urukthapelstatin a

A cyclic thiopeptide-urukthapelstatin A is secreted by *Mechercharimyces asporophorigenens* YM11–542. The molecular formula and weight of urukthapelstatin are C_34_H_30_N_8_O_6_S_2_ and 733 Da, respectively. Some previous research have shown the anti-cancer roles of urukthapelstatin A by growth inhibition against breast cancer (MCF-7), lung cancer (A549, NCI-H460, and DMS-114), ovarian cancer (SK-OV3, OVCAR-8, OVCAR-5, OVCAR-4, and OVCAR-3) and colon cancer (HCT-116) [57].

### Bacteria as carriers for colorectal cancer therapeutic agents

**Fig. 3:**
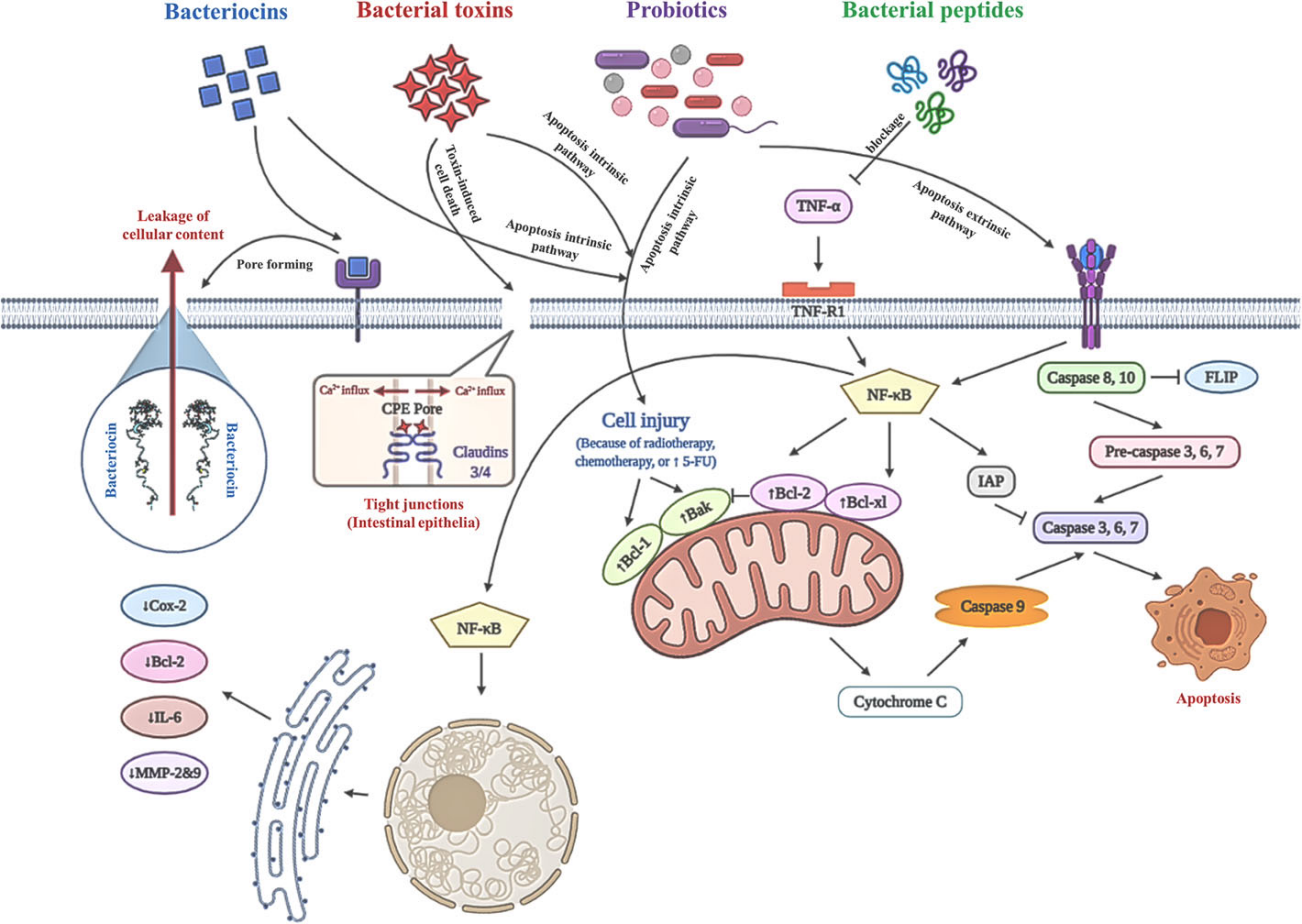
Some of the underlying molecular mechanisms for colorectal cancer bacteriotherapy. The whole cell bacteria (especially Lactic acid bacteria known as LABs) may lead cancer cells to apoptosis through intrinsic or extrinsic pathways. Bacterial peptides such as arenamides can prevent TNF-induced expression of pro-inflammatory mediators by NF-κB pathway blockage. Bacterial toxins such as *C. perfringens* enterotoxin (CPE) can directly interact with claudin-3 and claudin-4, which are over-expressed in colorectal cancerous cell membrane. This attachment results in pore formation through the membrane and cell death due to loss of cellular osmotic equilibrium. Another mechanism for bacterial toxins’ anti-cancer effect is cytotoxicity through apoptosis intrinsic pathway. Bacteriocins such as nisin are able to form membrane-pores when they bind to special type of cell surface receptors which cause leakage of cellular content and cell death. Besides pore formation activity, bacteriocins may act as apoptosis initiator through the intrinsic pathway [10, 16, 30, 50, 65, 68, 69]

While traditional cancer therapeutics are not capable of eliminating completely cancerous cells in anaerobic regions, bacteria as targeted delivery vectors are promising tools to carry anti-cancer genes or anti-tumor medications to hypoxic areas in tumors [64]. Since tumors are in hypoxic regions, anaerobic bacteria can present in the tumor environments [65]. Bacteria have specific metabolic characteristics that enable them to invade tumor cells as a feature that can’t be reached by conventional all therapeutic agents [10]. For instance, some strains can release secondary bioactive metabolites that are synthesized by a complex of the enzyme called non-ribosomal peptide synthetases (NRPSs). These peptides have specific characteristics in their structures including N-terminally attached fatty acid chains, D-amino acids, N- and C-methylated residues, glycosylated amino acids and heterocyclic elements, as well as phosphorylated residues. Hence, using these biological gene vectors results in a minimized dose and reduced toxicity [66]. Here, some of the examples of bacteria that are utilized as a vector for the treatment of colorectal cancer are discussed. These bacteria can cross the intestinal mucosa and trigger systemic immunity and mucosal immunity. Therefore, virulence-attenuated bacteria are appropriate carriers for heterologous protein delivery, DNA vaccines and other molecules for vaccination or treatment goals due to the specific metabolic characteristics [67]. For instance, *Lysteria monocytogenes* as an intracellular microorganism has been utilized as a vector for anti-cancer vaccines. It passes from intestinal membranes and activates immune responses. For example, it triggers CD8 and CD4 T-cells activity against tumors. It has also been found to be safe in clinical trials [68]. A recent study indicated that using a model of hepatic metastasis of colorectal cancer, demonstrated that making use of *L. monocytogenes* as a vector for a cancer vaccine notably magnifies the anti-tumor activity [69]. *Salmonella typhimurium* is another promising bacterium used for the delivery of anti-cancer agents. It is compatible with both aerobic and anaerobic environments, so it homes in both large and small tumors and activities innate or acquired immune response against tumors by activating of Toll-Like receptors (TLR) signaling pathway. TLR recognize the conserved component of *Salmonella typhimurium* which known as PAMPs (pathogen-associated molecular patterns) [70, 71]. In addition, administration of *S. typhimurium* systematically induces inflammasome pathways as bacteria colonize in the tumor area. The inflammasomes activate caspase-1 which cleavage pro-IL-1β and pro-IL-18 to produce active IL-1β and IL-18. On the whole, systemic administration of the bacteria induces pro-inflammatory cytokines production and activates the immune cells in the tumor site. *S. typhimurium* is a commonly used vaccine vector. Vector for 4-1BB ligand (4-1BBL) which is a DNA-based vaccine that successfully suppresses colorectal cancer progress in rats by triggering T cell-mediated immunity. Another example is *E. coli* Nissle 1917, an intestinal probiotic which has been used as a vector to deliver p53 and Tum-5 protein to tumor regions [64]. Engineered EcN is utilized to carry a genomic luxCDABE cassette holding a highly expressed lacZ vector in a murine model of colorectal cancer. EcN quickly localized in the gastrointestinal tract and colonized within the metastatic tumors, not in healthy organs [72]. Finally, *Clostridium novyi*-NT is a liposomal drug deliverer such as Doxorubicin to colon tumor cells [73] (Table 4). The potential of *C. novyi*-NT to modulate the tumorous area leads it to be a promising carrier for chemotherapeutic agents that target colorectal cancer cells. Several examinations illustrated that some therapeutic agents can decrease immune suppression, which is caused by tumors. For example, Doxorubicin, anti-metabolites gemcitabine and 5-FU can decrease the number of MDSCs. therefore, they can diminish immune suppression. Thereupon, delivering 5-FU with *C. novyi*-NT seems to be a rational and highly effective method to combat CRC cells [77, 78]. Some of the molecular mechanisms in bacterial therapy are shown in Fig. 3.

**Table 4:** Bacteria as carriers for colorectal cancer therapeutic agents

Microorganism	Gram Information	Combined agent	References
*Lysteria monocytogenes*	Gram-positive	Anti-cancer vaccines	[68]
*Salmonella typhimurium*	Gram-negative	Cytokines like IL-2, CCL21, Cytotoxic proteins, enzymes, vaccines	[71, 74–76]
*E.coli* Nissle 1917	Gram negative	P53 and Tum-5 protein	[64]
*Clostridium* novyi-NT	Gram-positive	Liposomal doxorubicin	[73]

### Limitations of bacteriotherapy methods

As previously discussed, bacteriotherapy involves the use of bacteria or their products to treat diseases and generally involves the use of probiotics, fecal matter transplantation (FMT) or intestinal microbiota transplantation (IMT), and synbiotics [79], and each in turn, has advantages and disadvantages that have attracted the attention of researchers. For example, in the FMT method in which a healthy person’s liquid stool suspension is injected into a patient, are some limitations that include the cost of the technique, the availability of stool donors, and the time of screening. Therefore, researchers have been able to overcome this limitation in the treatment of colorectal cancer by using frozen feces [80–82]. Numerous studies have shown that the combination of bacteriotherapy methods with different therapeutic approaches in the treatment of various cancers, especially colorectal cancer, has limitations. In this regard, researchers have mentioned limitations in combining chemotherapy and bacteriotherapy methods, such as incorrect targeting of the tumor, lack of proper penetration into the target tissue, and limited toxicity of cancer cells [83, 84]. Therefore, in general it can be said that although bacteria can be used as anti-tumor agents, however, it has major limitations including bacterial toxicity, DNA instability and limited targeting efficiency, lack of selection of appropriate, safe bacterial strain and lack of accompaniment with other therapeutic approaches [85, 86].

## Conclusion

Death and relapse rates and higher occurrence of a different kind of cancer reveal us that conventional cancer therapies are not effective enough and cause numerous side effects on the cancer patients. Therefore, nowadays researchers are concentrating on other approaches such as bacteriotherapy as a novel effective therapeutic method with fewer or with no side effects to pay the way of cancer therapy [87]. Several applications of bacterial agent have been developed by taking into consideration of their special features for tumor targeting such as post-administration control, specific internalization into the cancer cells, having specific toxicity against cancer cells, great cytotoxic activity, easy design and modification [87, 88]. Despite these special features of bacteria using for bacteriotherapy the drawbacks of this method cannot be overlooked; including the short life of bacterial peptide, innate bacterial toxicity, and unstable DNA [89]. It seems that *L. monocytogenes* strain induced an effective anti-tumor T-cell response, therefore provide it can be used in vaccine development for CRC patients with liver metastasis [90]. In addition, *S. typhimurium* declined the tumor size and improves the survival in a mouse model of CRC via increasing the hepatic NK cells [91].

n regard to pons and cons of this approach, most research on bacteriotherapy have been ending in the in vitro phase and only a few ones have gone from the in vitro condition to the clinical trial due to possible uncontrolled complications and side effects which mean that there is still room for exploring further [92, 93]. Since, CRC is a multifactorial disease, a single treatment cannot destroy the tumor and a combination of new and diverse treatments is necessary. By the way, bacterial therapy is combined with traditional methods, has been effectively used to treat CRC patients with positive results. Bacterial therapy of CRC using peptides and toxins is a talented method to save the lives of numerous CRC patients. Nonetheless, further surveys are necessary to improve the effectiveness of this novel strategy to use it in medical practice without any more harmful effects on patients’ health.

## Data Availability

The datasets used and/or analyzed during the current study are available from the corresponding author on reasonable request.

## Abbreviations

CRC: Colorectal cancer
IBD: Inflammatory bowel disease
5-FU: 5-Fluorouracil
CDTs: Cytolethal distending toxins
Cif: Cycle inhibiting factor
CEA: Carcinoembryonic antigen
CNF: Cytotoxic necrotizing factor
DT: Diphtheria toxin
T_reg_: Regulatory T cells
STm: *Salmonella enterica* serovar Typhimurium
STm^ΔaroA^: Aromatase A-deficient STm
TNF-α: Tumor necrosis factor α
Entap: Enterococcal anti-proliferative peptide
NRPs: Nonribosomal Peptides
NRPSs: Non-ribosomal peptide synthetases
TLR: Toll-Like receptors
PAMPs: Pathogen-associated molecular patterns
4-1BBL: Vector for 4-1BB ligand
FMT: Fecal matter transplantation
IMT: Intestinal microbiota transplantation
